# An Intervention Study Assessing the Effects of a Breast and Cervical Cancer Health Education Program on Knowledge, Attitudes, and Practices Among Adolescent Female Students in Madurai, South India

**DOI:** 10.7759/cureus.103131

**Published:** 2026-02-06

**Authors:** Trupti Bodhare, Samir Bele, Shalini V, Santha Sheela Kumari K, Gavin Francis J, Bairavan Mangaiyarkarasi Meenakshi, Salmathul Jaseela

**Affiliations:** 1 Community and Family Medicine, All India Institute of Medical Sciences, Madurai, IND; 2 Community Medicine, Velammal Medical College Hospital and Research Institute, Madurai, IND

**Keywords:** attitudes, breast cancer, cervical cancer, health education, knowledge, practice

## Abstract

Background

In India, the incidence of breast and cervical cancer is alarming, with inadequate screening rates highlighting the need for improved awareness and practices to combat late-stage diagnoses. Health education plays a crucial role in promoting effective cancer prevention and detection practices, especially among young female students, as early intervention fosters enduring health-conscious behaviors.

Methodology

In a quasi-experimental study conducted from October 2024 to April 2025, 250 female students from classes 9-12 at a private school in Madurai received health education. A semi-structured questionnaire collected socio-demographic data and assessed breast and cervical cancer knowledge, attitudes, and behaviors, which were examined immediately and three months after the intervention.

Results

The mean age of the students was 14.63 ± 1.09 years; 211 (84.4%) belonged to the upper socioeconomic class, and 246 (98.4%) had experienced menarche. Three months post-intervention, breast cancer knowledge scores increased from 28.52 ± 21.84 to 78.50 ± 21.57 (t = 25.40, p < 0.01), and attitude scores improved from 78.86 ± 10.03 to 85.92 ± 9.87 (t = 8.08, p < 0.01). Similarly, the overall knowledge for cervical cancer increased from 27.69 ± 20.89 to 79.47 ± 22.73 (t = 25.92, p < 0.01), and the overall attitude toward cervical cancer improved from 66.61 ± 9.50 to 81.98 ± 8.82 (t = 18.54, p < 0.01). After three months, 87 (34.8%) of participants promoted breast self-examination (BSE), and 72 (28.8%) promoted cervical cancer screening.

Conclusions

The structured health education program effectively enhanced schoolgirls' understanding and practices regarding breast and cervical cancer. However, sustained behavior changes require ongoing support, highlighting the importance of continuous reinforcement.

## Introduction

Breast and cervical cancers rank among the leading causes of cancer-related morbidity and mortality in women worldwide [1,2]. Globally, 2.3 million new breast cancer cases and 670,000 deaths occurred in 2022 [1,3]. The lifetime risk of developing breast cancer varies by development level, with 1 in 12 women affected in high Human Development Index (HDI) countries compared to 1 in 27 in low HDI nations [1]. Key risk factors include high body mass index, alcohol and tobacco use, elevated fasting plasma glucose, lack of physical activity, and unhealthy dietary patterns.

With an estimated 660,000 new cases and 350,000 deaths worldwide in 2022, cervical cancer is the fourth most frequent malignancy in women [2,3]. Cervical cancer is the leading killer, particularly in low- and middle-income nations [2]. HIV co-infection enhances the risk [2], and nearly all cases are associated with high-risk HPV infection. In 2021, hazardous sexual behaviors were responsible for 9.9 million disability-adjusted life years (DALYs) owing to cervical cancer, with smoking listed as a secondary cause [3]. Cervical cancer is prevalent in areas where screening and immunization are not widely available, particularly in Southeast Asia, Central America, and Sub-Saharan Africa [2,3].

In India, the incidence of breast cancer among women is 26.6%, with 192,020 reported cases, while cervical cancer represents 17.7% of all cancer cases, totalling 127,528 cases in 2022 [4]. Alarmingly, the National Family Health Survey-5 (NFHS-5) fact sheet reveals that only 1.9% of women have undergone cervical cancer screening, and a mere 0.9% have received a breast cancer examination [5].

Despite advancements in early detection and treatment, late-stage cancer diagnoses continue to pose a major challenge, often due to a lack of awareness, misconceptions, and poor screening practices [6,7].

Recent studies from South India indicate limited awareness and low screening rates for breast and cervical cancer. A community-based study conducted in Chengalpattu, Tamil Nadu, revealed inadequate awareness and engagement in breast cancer screening, suggesting that there must be improved health education initiatives [8]. Furthermore, urban women demonstrated low rates of cervical cancer screening and awareness, highlighting a critical area for enhancement through targeted educational programs [9]. Research conducted among students also indicates varying levels of knowledge, implying that even educated youth exhibit gaps in their comprehension of these essential health issues [10].

Structured health education initiatives in several countries have improved breast and cervical cancer knowledge, attitudes, and practices. Educational programs in India have increased cervical cancer awareness and vaccination intentions, according to numerous research studies. A behavioral-change education program in rural Tamil Nadu significantly increased breast cancer awareness and self-examination [11,12]. These findings demonstrate the importance of focused health education in worldwide cancer awareness and prevention.

Young female students are a key demographic for targeted educational interventions, as instilling awareness at an early age can lead to sustained health-conscious behaviors [13]. However, there are limited studies on school-based, multi-component health education programs in India that have assessed comprehensive knowledge, attitude, and practice (KAP) changes for breast and cervical cancer or measured sustainability over time. This study investigates the changes in awareness and health-seeking behaviors among female students in Madurai before and after an educational intervention. Key objectives include assessing the knowledge, attitudes, and practices regarding breast and cervical cancer prevention, measuring the intervention's effectiveness, and examining the participation of female adolescents in BSE, as well as their roles as health advocates in their communities.

## Materials and methods

Study design and study setting

A quasi-experimental study was conducted among female adolescent students from classes 9 to 12 at a private school in Madurai from October 2024 to April 2025, using convenience sampling.

Participants and eligibility criteria

There are a total of 471 female students in classes 9 to 12 The requisite sample size for a finite population of 451 (excluding 20 students who were involved in pilot study), with a 5% absolute error and a 95% confidence level, was 208, calculated using the formula n = [N Z² p (1-p)] / [d² (N-1) + Z² p(1-p)]. According to the results of the pilot study, the anticipated proportion was almost 50%. To account for a 20% attrition rate, the final sample size was set at 250 participants. All 451 female students from 9 to 12th standards were enrolled for the baseline assessment, and a simple random sampling method was employed to choose 250 students from a population of 451 females via a random number generator. Students who were unwilling to participate, lacked parental consent, and were absent during baseline data collection or at the time of first intervention were excluded. Only participants with complete data were included in the final analysis.

Data collection tool

A semi-structured, self-administered questionnaire was employed and had three parts. The first part asked for socio-demographic information. The second part asked 13 questions about breast cancer risk factors, symptoms, and prevention, as well as 8 questions about attitudes toward prevention and screening. The third part asked 9 questions about cervical cancer knowledge and 7 questions about attitudes. Knowledge was categorized as “No,” “Don’t know,” and “Yes,” while attitudes were measured on a 5-point Likert scale. For knowledge items, accurate answers received a score of 1, while incorrect or "don't know" responses received a score of zero. Attitude items were evaluated using a 5-point Likert scale (1 = strongly disagree to 5 = strongly agree), where higher scores reflect more favorable attitudes. For items with negatively worded attitudes, the scoring was changed so that higher scores always meant more positive attitudes. The practice items were evaluated according to actual behaviors, with responses classified to indicate the implementation of the suggested practices. Total scores were computed for each domain, with elevated scores indicating enhanced knowledge, more favourable attitudes, or suitable practices. Breast cancer knowledge scores were from 0 to 13, while breast cancer attitude scores varied from 8 to 40. Scores for cervical cancer knowledge and attitude varied from 0 to 9 and 7 to 35, respectively. The questionnaire was created based on relevant literature. Experts reviewed it to validate its content and ensured that it comprehensively covers all critical areas related to knowledge, attitudes, and practices.

Pilot study

A pilot study involving 20 female adolescent students assessed the feasibility of the study design and validated the data collection tool. The questionnaire showed good reliability with a Cronbach’s alpha of 0.8. Minor revisions were made based on feedback, and participants from the pilot study were excluded from the main study.

Intervention

The study comprised four phases: First, a baseline assessment evaluated students' knowledge, attitudes, and practices regarding breast and cervical cancer. Next, a structured health education intervention was conducted. The health education intervention comprised four structured sessions, each lasting 60 to 90 minutes. The sessions included a range of subjects related to breast and cervical cancer, encompassing anatomy, epidemiology, risk factors, symptoms, screening, immunization, prevention, and therapy. Various educational methods were employed, including interactive lectures, models, diagrams, videos, group discussions, and question-and-answer sessions. One-week post-intervention, the immediate effects were evaluated by re-administering the same questionnaire to assess changes in knowledge and attitudes. Finally, researchers collected data three months later to determine the program's effects over a longer period, which allowed for an evaluation of sustained knowledge and behavioural changes among participants.

Ethical considerations

Ethical clearance was obtained from the Institutional Ethical Committee (Ref: VMCIEC/065/2024), and permission was secured from the school administration. Written informed assent was obtained from participants, along with parental consent.

Data management and statistical analysis

Data were entered into Google Excel sheets and analyzed using Jamovi software (version 2.6.44) (https://www.jamovi.org). Descriptive statistics were applied to summarize baseline characteristics. Changes in knowledge and attitudes between pre- and post-intervention were assessed using the paired t-test, while practices related to breast and cervical cancer were evaluated using the Chi-square test/ Fisher’s exact test. A p-value of <0.05 was considered statistically significant.

## Results

The study included 250 female school students aged between 13 and 17 years, with a mean age of 14.63 ± 1.09 years. Most of them belonged to the Hindu religion 231 (92.4%), resided in urban areas 220 (88%), and were from nuclear families 203 (81.2%). Socioeconomic status, assessed using the BG Prasad scale, showed that 211 (84.4%) of students belonged to the upper class. Regarding health status, 146 (58.4%) of participants had a normal BMI, while 84 (33.6%) were underweight. Almost all students (98.4%) had attained menarche, with a mean age of 12.19 ± 0.97 years. Around 20 (8%) of the participants reported a family history of cancer (Table 1).

**Table 1:** Socio-demographic characteristics of the study participants.

Variable	Category	Frequency (n=250)	Percentage (%)
Age	< 14	44	17.6%
	14 -16	204	81.6%
	> 16	2	0.8%
Religion	Hindu	231	92.4%
	Muslim	11	4.4%
	Christian	8	3.2%
Place of residence	Urban	220	88%
	Rural	30	12%
Type of family	Nuclear family	203	81.2%
	Three-generation family	37	14.8%
	Joint family	10	4%
Socioeconomic status	Upper class	211	84.4%
	Upper middle class	32	12.8%
	Middle class	7	2.8%
Body Mass Index	Underweight	84	33.6%
	Normal	146	58.4%
	Overweight	16	6.4%
	Obese	4	1.6%
History of family members or relatives having cancer	Yes	20	8%
	No	230	92%

Table 2 shows that breast and cervical cancer knowledge, attitudes, and practices improved statistically after the health education intervention. The intervention significantly increased knowledge of breast cancer risk factors, with a mean increase from 37.40 ± 28.72 to 84.20 ± 20.91 (t = 22.32, p < 0.01). Signs and symptoms knowledge increased from 26.53 ± 24.14 to 91.33 ± 16.78 (t = 36.85, p < 0.01), whereas prevention and early detection knowledge improved from 20.67 ± 27.13 to 87.87 ± 22.93 (t = 30.50, p <0.01) Improvement in overall knowledge score from 28.52 ± 21.84 to 88.34 ± 15.48 (t = 37.86, p < 0.01). Attitude scores improved significantly: preventive attitude increased from 80.56 ± 14.78 to 94.00 ± 10.30 (t = 12.86, p < 0.01), screening attitude from 73.56 ± 14.55 to 92.88 ± 10.63 (t = 17.65, p < 0.01), and health-seeking intentions from 80.66 ± 12.35 to 85.40 ± 11.76 (t = 4.74, p < 0.01). The overall attitude improved from 78.86 ± 10.03 to 89.42 ± 8.63 (t = 13.72, p < 0.01).

**Table 2:** Comparison of pre- and immediate post-intervention knowledge and attitude scores using paired t-test (n=250)

Variable	Category	Breast Cancer			Cervical Cancer		
		Mean and Standard Deviation	t value	p value	Mean and Standard Deviation	t value	p value
Knowledge towards risk factors	Pre-intervention	37.40 ± 28.72	22.32	<0.01	37.04 ± 25.77	30.91	<0.01
	Post-intervention	84.20 ± 20.91			95.68 ± 14.13		
Knowledge towards signs and symptoms	Pre-intervention	26.53 ± 24.14	36.85	<0.01	14.60 ± 27.93	37.43	<0.01
	Post-intervention	91.33 ± 16.78			93.60 ± 18.45		
Knowledge towards prevention and early detection	Pre-intervention	20.67 ± 27.13	30.50	<0.01	17.40 ± 30.18	35.67	<0.01
	Post-intervention	87.87 ± 22.93			95.00 ± 16.31		
Overall knowledge	Pre-intervention	28.52 ± 21.84	37.86	<0.01	27.69 ± 20.89	43.73	<0.01
	Post-intervention	88.34 ± 15.48			95.07 ± 11.99		
Preventive attitude	Pre-intervention	80.56 ± 14.78	12.86	<0.01	70.12 ± 14.38	16.06	<0.01
	Post-intervention	94.00 ± 10.30			88.00 ± 11.72		
Screening attitude	Pre-intervention	73.56 ± 14.55	17.65	<0.01	67.87 ± 13.39	15.36	<0.01
	Post-intervention	92.88 ± 10.63			86.56 ± 13.79		
Health seeking intentions	Pre-intervention	80.66 ± 12.35	4.74	<0.01	61.20 ± 13.65	11.93	<0.01
	Post-intervention	85.40 ± 11.76			76.84 ± 15.75		
Overall attitude	Pre-intervention	78.86 ± 10.03	13.72	<0.01	66.61 ± 9.50	20.65	<0.01
	Post-intervention	89.42 ± 8.63			84.19 ± 9.62		

Cervical cancer knowledge improved after the intervention. Risk factor comprehension increased from 37.04 ± 25.77 to 95.68 ± 14.13 (t = 30.91, p < 0.01), signs and symptoms awareness from 14.60 ± 27.93 to 93.60 ± 18.45 (t = 37.43, p < 0.01), and preventative knowledge from 17.40 ± 30.18 to 95.00 ± 16.31 (t = 35.67, p < 0.01). Overall knowledge rose from 27.69 ± 20.89 to 95.07 ± 11.99 (t = 43.73, p < 0.01). Attitudes improved significantly: preventive attitude rose from 70.12 ± 14.38 to 88.00 ± 11.72 (t = 16.06, p < 0.01), screening attitude from 67.87 ± 13.39 to 86.56 ± 13.79 (t = 15.36, p < 0.01), and health-seeking intentions from 61.20 ± 13.65 to 76.84 ± 15.75 (t = 11.93, p < 0.01). Overall attitudes towards cervical cancer increased (t = 20.65, p < 0.01) from 66.61 ± 9.50 to 84.19 ± 9.62.

Table 3 reveals that, three months following the intervention, knowledge and attitudes regarding breast cancer remained significantly increased compared to pre-intervention levels, albeit slightly lower than the immediate post-intervention scores. Specifically, the overall knowledge score was 78.50 ± 21.57 (t = 25.40, p < 0.01), while the overall attitude changed to 85.92 ± 9.87 (t = 8.08, p < 0.01). Notably, health-seeking intentions exhibited no significant long-term change (pre = 80.66 ± 12.35, post = 81.70 ± 11.04, t = 1.01, p = 0.315), suggesting a potential need for reinforcement strategies. Additionally, there was a sustained improvement in knowledge and attitudes toward cervical cancer. Overall knowledge remained high at 79.47 ± 22.73 (t = 25.92, p < 0.01), and overall attitude was recorded at 81.98 ± 8.82 (t = 18.54, p < 0.01). Although there was a marginal increase in health-seeking intention scores, this difference was statistically significant (68.68 ± 14.09, t = 6.07, p < 0.01).

**Table 3:** Comparison of pre- and three months post-intervention knowledge and attitude scores using paired t-test (n=250).

		Breast Cancer			Cervical Cancer		
Variable	Category	Mean and Standard Deviation	t value	p value	Mean and Standard Deviation	t value	p value
Knowledge towards risk factors	Pre-intervention	37.40 ± 28.72	11.09	<0.01	37.04 ± 25.77	21.81	<0.01
	Post-intervention	68.20 ± 31.90			84.40 ± 21.99		
Knowledge towards signs and symptoms	Pre-intervention	26.53 ± 24.14	30.42	<0.01	14.60 ± 27.93	19.05	<0.01
	Post-intervention	86.53 ± 20.88			73.60 ± 38.59		
Knowledge towards prevention and early detection	Pre-intervention	20.67 ± 27.13	20.61	<0.01	17.40 ± 30.18	18.10	<0.01
	Post-intervention	76.13 ± 32.23			73.00 ± 36.69		
Overall knowledge	Pre-intervention	28.52 ± 21.84	25.40	<0.01	27.69 ± 20.89	25.92	<0.01
	Post-intervention	78.50 ± 21.57			79.47 ± 22.73		
Preventive attitude	Pre-intervention	80.56 ± 14.78	6.63	<0.01	70.12 ± 14.38	12.57	<0.01
	Post-intervention	88.60 ± 13.20			86.16 ± 13.72		
Screening attitude	Pre-intervention	73.56 ± 14.55	14.86	<0.01	67.87 ± 13.39	16.93	<0.01
	Post-intervention	91.68 ± 13.40			88.05 ± 12.51		
Health seeking intentions	Pre-intervention	80.66 ± 12.35	1.01	0.315	61.20 ± 13.65	6.07	<0.01
	Post-intervention	81.70 ± 11.04			68.68 ± 14.09		
Overall attitude	Pre-intervention	78.86 ± 10.03	8.08	<0.01	66.61 ± 9.50	18.54	<0.01
	Post-intervention	85.92 ± 9.87			81.98 ± 8.82		

Practice-related data show significant behavioral changes after a health education intervention. Post-intervention BSE rates rose from 10 (4%) to 181 (72.4%), but they dropped to 109 (43.6%) after three months, indicating the need for continued reinforcement. Physical activity participation (≥150 minutes/week) increased from 80 (32%) to 231 (92.4%) post-intervention, but decreased to 171 (68.4%) long-term. Twenty-four percent promoted BSE post-intervention, and 87 (34.8%) after three months. Post-intervention cervical cancer screening promotion rose to 72 (28.8%) after three months. Statistical analysis showed a substantial difference (p < 0.01) in percentages before and after the intervention (Table 4). 

**Table 4:** Practice towards breast and cervical cancer among participants: Pre-intervention, immediate, and 3 months post-intervention (n=250). BSE: Breast-self-examination

Variable	Category	Frequency n (%)	Chi square/Fisher’s exact (p value)
Number of students who practiced BSE	Pre-intervention	10 (4)	245.7 (<0.01)
	Immediate post-intervention	181 (72.4)	
	3 months post-intervention	109 (43.6)	
Number of students who have engaged in physical activity for at least 150 minutes per week	Pre-intervention	80 (32)	201.36 (<0.01)
	Immediate post-intervention	231 (92.4)	
	3 months post-intervention	171 (68.4)	
Number of students who have promoted BSE to their households, friends and neighbours	Pre-intervention	0	100.73 (<0.01)
	Immediate post-intervention	61 (24.4)	
	3 months post-intervention	87 (34.8)	
Number of students who have promoted cervical cancer screening to their households, relatives and neighbours	Pre-intervention	0	82.94 (<0.01)
	Immediate post-intervention	62 (24.8)	
	3 months post-intervention	72 (28.8)	

## Discussion

This study assessed an educational intervention's effectiveness in enhancing awareness, modifying perceptions, and promoting proactive health-seeking behaviors among adolescent female school students. The participants in our study were early to mid-adolescents, which supports the rationale for starting preventive education before adulthood. This is especially important in relation to health-seeking behavior, in contrast to the older cohorts studied by Lata et al. [14]. The study population was predominantly from the upper socioeconomic class, a finding attributed to the private school setting and contrasting significantly with studies like Potluri et al., which included larger proportions from middle and lower classes [15]. A family history of cancer was reported by 8% of participants, closely aligning with the 7% found by Potluri et al. [15]. The study found that around one-third of participants were underweight, which is significantly lower than the thinness reported among adolescent girls in NFHS-5, indicating a relatively better nutritional status [5]. Furthermore, the study found that most participants had attained menarche earlier than what was reported in the NFHS pooled data [16]. The distinctions in study populations, settings, and data collection methods may lead to similarities or differences in baseline characteristics across studies.

This study demonstrated a significant rise in the overall knowledge score about breast cancer among the participants. Particular domains of knowledge, such as risk factors, signs and symptoms, and early detection, exhibited similar significant enhancements three months post-intervention. These results are similar to studies by Lata et al., which found that nursing students' knowledge increased from before to after an educational program, and Gadpande et al., which showed that reproductive-aged women also had more knowledge after an educational intervention [14,17]. The larger improvement in the knowledge of the study's adolescent cohort shows that younger participants were more receptive to structured educational content.

After the intervention, there was a significant improvement in attitudes towards breast cancer prevention and screening, and these positive attitudes remained largely intact after three months, significantly exceeding the baseline. The improvement noted in this study exceeds the marginal attitude score change reported by Lata et al. [14]. In contrast, Gupta et al. observed significant attitude changes in the 20-30 age group [18]. The differences may stem from regional variations and disparities in the sociodemographic and educational status of the participants.

BSE practice surged immediately post-intervention but fell to around half of the participants practicing BSE after three months. Our findings are consistent with those obtained by Ogunmodede et al., who demonstrated an improvement in their knowledge and practice of BSE after using an intervention tool [19]. Similarly, the study by Lata et al. showed a practice score improvement of up to 86.81% among nursing students [14]. Encouragingly, about one-fourth of participants promoted BSE to peers, family members, and relatives, highlighting the potential of school-based health education to foster community awareness. Integrating adolescent health education into school curricula can significantly enhance community awareness, leveraging peer networks to create a powerful ripple effect that promotes overall health and well-being.

The study indicates that adolescents with minimal prior knowledge of BSE can adopt preventive behaviors when provided with information. However, a decrease in sustained behavior suggests the necessity of ongoing follow-up interventions.

In the domain of cervical cancer, the educational intervention yielded even more pronounced improvements in knowledge than those observed for breast cancer. Notably, awareness of risk factors and prevention methods saw especially high gains, with post-intervention scores nearing 95%. These findings align with research by Mary et al., which indicated improved cervical cancer knowledge in adolescent girls following an educational session [20]. Additionally, Samah Abd Elhaliem Said et al. observed a substantial increase in women with good knowledge, from 10.8% pre-intervention to 64.6% post-intervention, underscoring the effectiveness of structured educational programs [21].

Attitudes regarding cervical cancer prevention and screening have markedly improved, along with intentions to seek healthcare services. Overall attitude scores also showed significant improvement. These positive changes were important, but not as strong as those seen for breast cancer. This variation could be because there is still a lot of cultural stigma and misunderstanding about cervical cancer and its connection to sexual transmission.

Three months post-intervention, knowledge and attitude towards cervical cancer remained significantly improved from baseline. While these metrics slightly decreased from immediate post-intervention levels, mirroring breast cancer data trends suggesting knowledge decay without reinforcement, the sustained improvements indicate the intervention's positive medium-term efficacy.

The study's limitations highlight challenges in generalizing findings beyond the specific private school context in Madurai, the lack of a control group affecting causal attribution, and the uncertainty of long-term behavior change. Self-reported practices, such as BSE, were not objectively verified, introducing potential social desirability bias. Furthermore, HPV vaccination uptake and cervical cancer screening practices were limited by factors like age, parental consent, and access, which restricted a more comprehensive evaluation.

## Conclusions

A structured health education program positively impacted adolescent schoolgirls' knowledge, attitudes, and self-reported practices concerning breast and cervical cancer. Periodic reinforcement and the incorporation of cancer awareness programs into the regular school health curriculum may be necessary for sustained behavioral change. The study suggests school-based health education can foster early preventive behaviors and empower adolescents as health promoters. Future research should aim for long-term impact assessments and better integration with preventive health services to ensure lasting benefits.

## Appendices

Questionnaire- Assessment of Knowledge, Attitude and Practice on Breast and Cervical Cancer Among School Students

Instructions to the Student

• This questionnaire has no right or wrong answers. • Do NOT write your name. Your responses are anonymous. • Please tick (✔) the correct option. • Answer all questions honestly. • Time required: 15 minutes

Domain 1 Socio-demographic details

1.1, Age in completed years: 1.2, Class/Standard that you are studying: 1.3, Residence: Urban/ Rural 1.4, Type of family: 1.5, Total family members: 1.6, Total Family Income: 1.7, Have you attained menarche: (Yes/No)? 1.8, If yes, what is the age at which you attained menarche: 1.9, Height in cm: 1.10, Weight in kg: 1.11, History of Family members or relatives having cancer:

Domain 2 knowledge, attitude and practice towards breast cancer 2.Knowledge of breast cancer (2.1 - 2.13) Yes/No/Don’t know 2.1, Do you think early age (before 9 years of age) of attaining menarche (first menstrual cycle) is a risk factor for breast cancer? 2.2, Do you think late menopause (after 55 years of age) could be a risk factor for breast cancer? 2.3, Do you think obesity could be a risk factor for breast cancer? 2.4, Do you think Physical inactivity could be a risk factor for breast cancer? 2.5, Do you think a mass over your breast could be a sign of breast cancer? 2.6, Do you think a mass under your armpit could be a sign of breast cancer? 2.7, Do you think puckering or dimpling of your breast skin could be a sign of breast cancer? 2.8, Do you think abnormal bleeding/discharge from your nipple could be a sign of breast cancer? 2.9, Do you think changes in the size of your breast could be signs of breast cancer? 2.10, Do you think changes in the shape of your breast could be signs of breast cancer? 2.11, Does a women who gives birth between the age of 20 to 30 years have a lower chance of developing breast cancer ? 2.12, Do you think adequate breast feeding to the child helps in prevention of breast cancer? 2.13, Do you think self-breast examination will help us to identify breast cancer in the early stage?

Attitude (2.14 - 2.21) Strongly agree/Agree/Neutral/Disagree/Strongly disagree

2.14, Avoiding smoking and alcohol will reduce my cancer risk

2.15, Healthy life style can prevent breast cancer.

2.16, All women (after menstruation) should do self-breast examination once a month.

2.17, All women (after menstruation) should do clinical breast examination once a year.

2.18, If anyone has abnormal symptoms in breast, should consult a doctor immediately.

2.19, “what the doctor might find”, will stop me from going to the doctor?

2.20, I’m confident talking about my symptom with the doctor.

2.21, Breast cancer can be cured if someone diagnosed in early stage.

Practice: (2.22 - 2.29) 2.22, Do you practice breast self-examination? Yes No 2.23, If Yes, Frequency of practicing self-breast examination_______________. 2.24, Reasons for not practicing breast self-examination_________________. 2.25, Do you (or) Will you practice clinical breast examination Yes No 2.26, If no, reason for not practicing clinical breast examination ________________. 2.27, Do you engage in physical activity for at least 150 minutes a week? 2.28, To how many households and neighbours have you taught about Breast self-examination? _____________. 2.29, To how many households, friends, family and neighbours you promoted breast cancer screening? ______________.

Domain 3 knowledge, attitude and practice towards cervical cancer 3.Knowledge of cervical cancer (3.1 - 3.9) Yes/No/Don’t know 3.1, Do you think Poor menstrual Hygiene is a risk factor for cervical cancer? 3.2, Do you think having multiple sexual partners is a risk factor for cervical cancer? 3.3, Do you think early age of sexual activity is a risk factor for cervical cancer? 3.4, Do you think Cigarette smoking is a risk factor for cervical cancer? 3.5, Do you think cervical cancer is due to the infection of Human Papilloma virus(HPV)? 3.6, Do you think bleeding in between periods could be a sign of cervical cancer? 3.7, Do you think Postmenopausal bleeding could be a sign of breast cancer? 3.8, Do you think cervical cancer can be screened in early stage using pap smear/VIA/VILI? 3.9, Do you know HPV vaccination can prevent cervical cancer?

ATTIUTE TOWARDS CERVICAL CANCER (3.10 - 3.16) Strongly agree/Agree/Neutral/Disagree/Strongly disagree 3.10, If my menstrual hygiene is good, there is a less chance of getting the cancer. 3.11, HPV vaccination at the age of 9-14 years can prevent cervical cancer 3.12, Screening (Pap smear/VIA/VILI) helps in prevention of cervical cancer. 3.13, Screening cause no harm to client 3.14, Screening is done once in every 3 years 3.15, I feel confident discussing reproductive health symptoms with a doctor 3.16 It is very important to consult doctor if there is a bleeding between periods.

PRACTICE TOWARDS CERVICAL CANCER: (3.17 - 3.21) YES/NO/MAY BE 3.17, Have you taken HPV vaccination? 3.18, If yes, how many doses have you taken? 3.19, If no, will you take HPV vaccination in future? 3.20, How many friends and sisters have you promoted to get vaccinated for HPV vaccine? 3.21, How many family household’s or neighbors have you promoted for screening cervical cancer?
